# Characterization of the seminal bacterial microbiome of healthy, fertile stallions using next-generation sequencing

**DOI:** 10.1590/1984-3143-AR2020-0052

**Published:** 2021-08-06

**Authors:** Carlota Quiñones-Pérez, Manuel Hidalgo, Isabel Ortiz, Francisco Crespo, José Luis Vega-Pla

**Affiliations:** 1 Laboratorio de Investigación Aplicada, Cría Caballar de las Fuerzas Armadas, Córdoba, España; 2 Veterinary Reproduction Group Department of Animal Medicine and Surgery, Universidad de Córdoba, Córdoba, España; 3 Centro Militar de Cría Caballar de Ávila, Cría Caballar de las Fuerzas Armadas, Ávila, España

**Keywords:** horse, microbiome, semen, next-generation sequencing

## Abstract

High-throughput sequencing studies have shown the important role microbial communities play in the male reproductive tract, indicating differences in the semen microbial composition between fertile and infertile males. Most of these studies were made on human beings but little is known regarding domestic animals. Seminal bacteria studies made in stallions mostly focus on pathogenic bacteria and on their impact on reproductive technology. However, little is known about stallion commensal seminal microflora. That ultimately hinders our capacity to associate specific bacteria to conditions or seminal quality. Therefore, the aim of this study was to characterize the seminal microbial composition of 12 healthy, fertile stallion using next-generation sequencing. Hypervariable region V3 was chosen for bacterial identification. A total of nine phyla was detected. The most abundant ones were Bacteroidetes (46.50%), Firmicutes (29.92%) and Actinobacteria (13.58%). At family level, we found 69 bacterial families, but only nine are common in all samples. Porphyromonadaceae (33.18%), Peptoniphilaceae (14.09%), Corynebacteriaceae (11.32%) and Prevotellaceae (9.05%) were the most representative ones, while the Firmicutes phylum displayed the highest number of families (23, a third of the total). Samples showed high inter-subject variability. Findings previously described in other species notably differ from our findings. Families found in human such as Lactobacillaceae, Staphylococcaceae and Streptococcaceae only represented a 0.00%, 0.17% and 0.22% abundance in our samples, respectively. In conclusion, Porphyromonadaceae, Prevotellaceae, Peptoniphilaceae and Corynebacteriaceae families are highly represented in the seminal microbiome of healthy, fertile stallions. A high variation among individuals is also observed.

## Introduction

Next-generation sequencing has been used in horses to characterize the bacterial flora of the digestive tract (Costa and Weese, 2012; Ericsson et al., 2016; Su et al., 2020; Daly et al., 2001). These studies brought to light the important role microbial communities play in maintaining the homeostasis of this complex environment not only in the horses (Costa and Weese, 2018) but also in other domestic animal species (Wu et al., 2016; Zhang et al., 2016; Rando, 2012; Ng et al. 2010). More recent studies have shown the interaction between bacterial flora and the host may contribute to the occurrence of laminitis (Milinovich et al., 2010; Al Jassim and Andrews, 2009), colic (Al Jassim and Andrews, 2009; Salem et al., 2019) and stomach ulcers (Al Jassim and Andrews, 2009). It has even described that they are able to induce alterations of behavioural and mood status in human beings (Costa and Weese, 2018; Goulet, 2015). These discoveries have led to the use of probiotics as a prophylactic and sometimes therapeutic tool for some digestive conditions in the horse (Coverdale, 2016; Swyers et al. 2008). These findings have opened the door to study the microbiome in new niches, such as lower respiratory tract (Manguin et al., 2020), conjunctive (LaFrentz et al., 2020) or female reproductive tract (Barba et al., 2020; Hou et al., 2013). However, little is known regarding the commensal flora of the male reproductive tract. That ultimately would hinder our capacity to associate specific bacteria to conditions.

There are very few studies of the male reproductive tract microbiome in humans (Hou et al., 2013; Liu et al., 2014; Weng et al., 2014) and practically none in animals (Rosenfeld et al., 2018; Javurek et al., 2016; Wickware et al., 2020; Serrano et al., 2020; Al-Kass et al., 2020). In spite of that, a few studies have already associated the presence of some bacteria families to fertility (Hou et al., 2013; Kiessling et al., 2008). In the studies performed in horses, researchers have mostly focused on the detection and reduction of pathogenic bacteria in the reproductive tract to prevent their spread (Samper, 2009; Al-Kass et al., 2019); whereas some others focused their research on associating bacteria genera to its effect on reproductive technologies (Moretti et al., 2009; Ortega-Ferrusola et al., 2009; Varela et al., 2018). To the best of our knowledge, there is only one article (Al-Kass et al., 2020) describing the seminal microbiome in horses. Results vary among those articles, maybe because microflora may depend on external factors, such as environment or region (Al-Kass et al., 2020). In order to have broader picture of the commensal flora of the stallion reproductive tract, more metagenomic analysis are needed. Therefore, the aim of this study is characterizing the seminal microbial composition of healthy, fertile stallion in the south of Spain using next-generation sequencing.

## Methods

### Ethical statement

Animals were raised and handled in accordance with the Spanish law for animal welfare (Law 32/07). Animals were not submitted to extra semen extractions for our experiment sample collection nor was their daily workflow interrupted. Samples were not collected for the purpose of the study.

### Animals and semen collection

#### Samples

Samples were obtained from 12 (seven Andalusian and five Arabian) healthy and fertile stallions located in the Equine Breeding Centre of the Spanish Army of Écija (Seville, Spain). Stallions ranged in age from 7 to 24 years and were included in the reproductive breeding program of the Centre. All the stallions were housed in individual boxes with a straw bedding. Faeces were removed from the housing daily. The feeding consisted of oats, commercial concentrate and water *ad libitum*. Animals lived under the same dietary and exercise conditions.

Semen was collected using an in-line gel-filter Missouri artificial vagina, with a mare in estrus as a teaser. An inner disposable plastic liner was used with each animal so to avoid cross-contamination. Semen was regularly collected, two or three times per week, in intervals of at least 24 h between semen collections (Monday, Wednesday and Friday) throughout the breeding season (from March to July). A total of 12 ejaculates were collected (one per stallion) by the end of the month of March. No clinical diseases were reported. All the stallions had physiological values of sperm quality parameters. Mean ± standard deviation values of the following parameters were: volume = 31.4 ± 21.2 ml; sperm concentration = 276.0 ± 95.1 spermatozoa/ml; total motility = 80.4 ± 7.9%; and progressive motility = 37.0 ± 10.1%.

#### DNA extraction control sample

A pattern was created in order to evaluate the quality of the DNA extraction and its amplification. It was composed of five field strain species. *Rhodococcus equi* and *Taylorella equigenitalis* came from the Microbiology Department of Military Veterinary Centre, (Ministry of Defence, Spain). The other three strain species came from Colección Española de Cultivos Tipo: *Staphylococcus aureus* (ATCC 43300), *Klebsiella pneumoniae* (ATCC 10031) and *Pseudomonas aeruginosa* (CECT 108).

The pattern sample contained 2x10^7^ bacteria, equally distributed among the five species (4x10^6^ cells each). Counting was performed with a Neubauer chamber. It was submitted to DNA extraction at the same time as the rest of samples.

### Next generation sequencing

#### DNA extraction

Samples were cryopreserved immediately after their extraction. Then, DNA extraction was performed using a ZymoBIOMICS® DNA Miniprep kit (Zymo Research, CA) commercial kit. Samples were previously submitted to a combination of mechanic and enzymatic-digestion cell disruption as described by Bag (Bag et al., 2016). Then, DNA extraction was performed following the manufacturer’s instructions.

#### Library preparation and sequencing:

Amplicons were obtained using an Ion 16S Metagenomics® kit (Thermo Fisher, Waltham, MA). This kit characterizes five different sets of 16S hypervariable regions, V2, V3, V4, V67 and V8. The library was constructed with an Ion Plus Fragment Library kit and amplicons were labelled with an XpressTM Barcode Adapters 1-16 kit. Samples were then pooled using Ion PGM® (Thermo Fisher, Waltham, MA), HiQ Sequencing kit®, Ion 316 v2 BC® chip and sequenced using the Ion 16S™ Metagenomics Workflow in Ion Reporter™ Software. OTUs were obtained from the Ion Reporter server system (Thermo Fisher Scientific, Waltham, MA, USA).

### Statistical analysis

The Ion Reporter server system was used for data analysis (Thermo Fisher Scientific, 2021). The α-diversity analysis with Chao 1 non-parametric model to confirm that all potential bacteria have been detected (Mira Obrador, 2014). OTUs from hypervariable region V3 was chosen for bacterial identification, as it obtained the highest number of copies. Moreover, it has been suggested to detect a wider range of bacterial species (Fullston et al., 2015). Then, mean values and standard error of the mean were calculated for each phylum. Inter-subject variability was calculated with Bray-Curtis dissimilarity index.

## Results

### Control

The microbiome is described at a taxonomic family level. The observed proportions in the pattern were 23.1% for Alcaligenaceae, 31.4% for Enterobacteriaceae, 15.5% for Pseudomonadaceae, 27.0% for Nocardiaceae, and 2.7% for Staphylococcaceae. In most of the cases, sequenced families have appeared in the expected proportion (20%). Nevertheless, Staphylococcaceae lowers its proportion (near 2.7%) in Enterobacteriaceae’s favour (31.4%).

### Microbial abundance and composition

**Figure 1:**
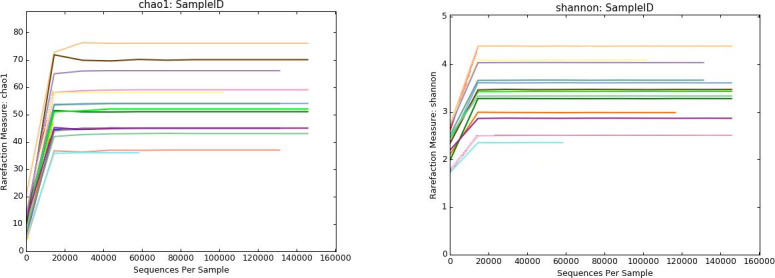
Rarefaction curves using Chao 1 model. All samples reach to the plateau, which is an indicator that all potential families have been detected.

**Figure 2:**
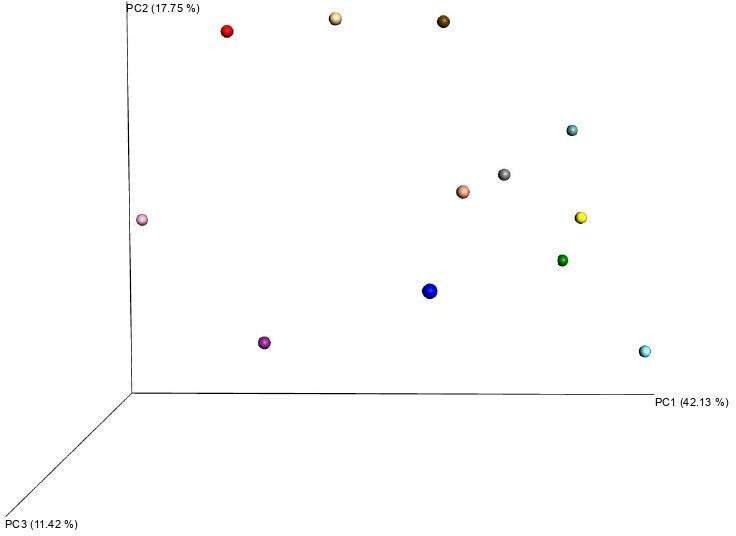
β-diversity using Bray-Curtis dissimilarity index. Samples are uniformly distributed along de spectrum. It was not possible to classify animals into subgroups, which is an indirect indicator of inter-subject variability.

Table 1 shows the number of valid sequences obtained and the number of which that were assigned to different OTUs. The α-diversity analysis show samples were sequenced to the plateau (Figure 1), which means that the analysis have virtually located all bacteria present in the samples (Espinosa, 2019). The β-diversity analysis shows a high inter-subject variability (Figure 2).

**Table 1:** Number of valid sequence (VS) and mapped sequences (MS) per sample according to V3 results. Numbers correspond to the stallions. Results are expressed as number of copies.

**Stallion**	**VS**	**MS**
1	113651	81924
2	101979	73542
3	142745	111842
4	57464	37400
5	22609	15790
6	60555	45921
7	80441	58957
8	58750	42277
9	53504	39195
10	101524	75815
11	78380	62325
12	76634	47306

A total of nine phyla were found. The most abundant ones were Bacteroidetes (46.50%), Firmicutes (29.92%) and Actinobacteria (13.58%). The following most common phyla were Fusobacteria (4.50%), Proteobacteria (4.32%) and Spirochaetes (4.10%), but only Proteobacteria was detected in all horses. Fusobacteria only appeared in five of them, and Spirochaetes in nine. The last three phyla were Synergestes (0.99%), Tenericutes (0.40%) and Chloroflexi (0.10%), which only were present in seven, two and one animals, respectively.

**Figure 3:**
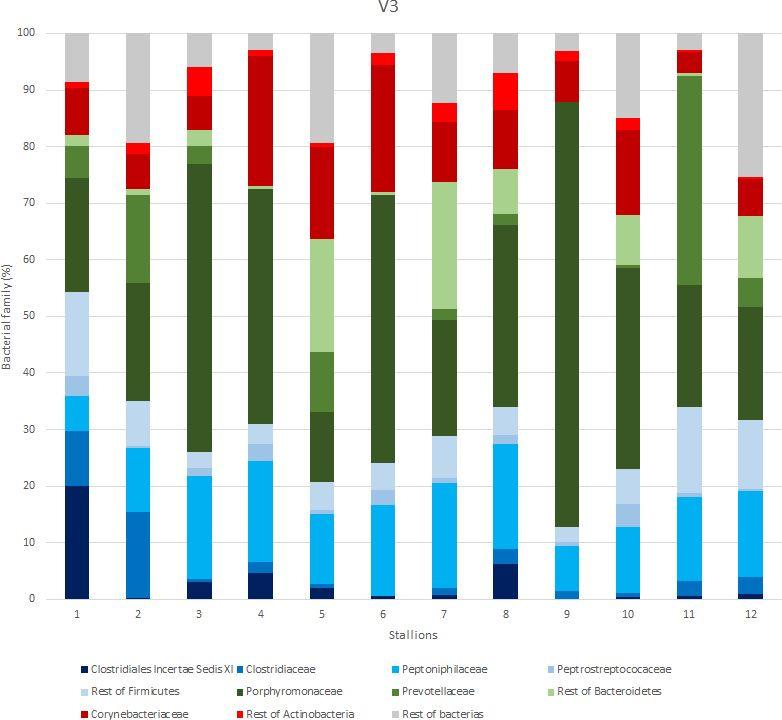
Detailed bacteria family composition of the samples. The results are expressed as percentages (%). Only common families with a relative abundance above 1% are included separately. Sections in blue represents Firmicutes phylum; green represents Bacteroidetes phylum; red represents Actinobacteria phylum; grey represents the rest of phyla.

**Figure 4:**
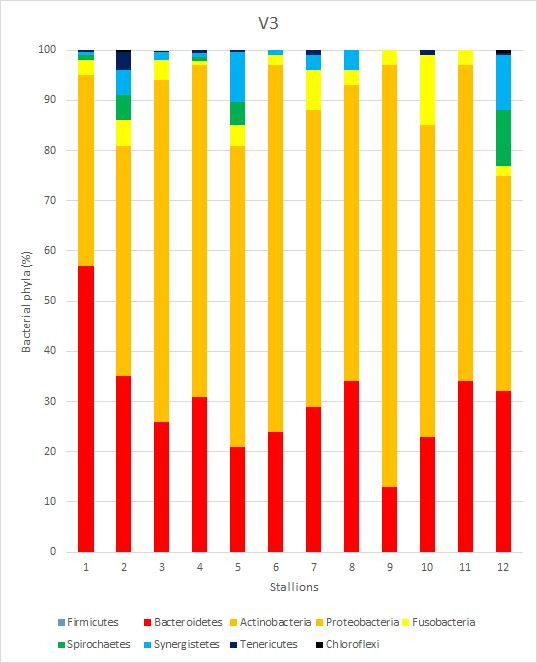
Phyla detected in samples. The results are expressed as percentages (%).

A total of 69 families (Figure 3) were found and nine phyla were found (Figure 4). Only 22 families out of 69 exceed a 1% presence. In addition, only nine appear to be common in all samples. The most common families were Porphyromonadaceae (32.61 ± 18.16%), Corynebacteriaceae (11.05 ± 6.10%), Peptoniphilaceae (13.69± 4.28%) and Prevotellaceae (10.05 ± 10.83%). The following most common families were Clostridiaceae (3.59 ± 4.22%), XI Family, which includes several non-identifiable genera of Clostridia (3.31 ± 5.60%) and Peptostreptococcaceae (3.05 ± 5.33%).

## Discussion

In this study, next generation sequencing has been used for characterizing the seminal microbiome of stallions. This technique is more efficient for analysing microbial flora than culture-based methods, especially for hard-to-cultivate species (Zhang et al., 2016). That is why it has been used for characterizing gut microbiome (Su et al., 2020), lower respiratory tract (Manguin et al., 2020), conjunctive (LaFrentz et al., 2020). To the best of our knowledge, there is only one paper analysing the seminal microbiome in stallions with NGS technology (Al-Kass et al., 2020). However, authors have stated that there may be variations due to external factors. (Wickware et al., 2020; Al-Kass et al., 2020; Tomaiuolo et al., 2020). Therefore, it is of the utmost interest to keep exploring seminal metagenomics.

According to our study, there are four main families that represent the seminal microbiome in healthy and fertile stallions: Porphyromonadaceae (Bacteroidetes phylum), Peptoniphilaceae (Firmicutes phylum), Corynebacteriaceae (Actinobacteria phylum) and Prevotellaceae (Bacteroidetes phylum). Our results greatly concur with the ones found in the north of Europe (Al-Kass et al., 2020). However, they plainly differ from studies made in human, where gram-positive bacteria prevail (Hou et al., 2013; Weng et al., 2014). With respect to other species, they also differ from mice (Rosenfeld et al., 2018; Javurek et al., 2016) and ram (Serrano et al., 2020).

Starting with the Bacteroidetes phylum, there is no a general consensus about its function in semen. It has been identified in healthy, fertile men (Hou et al., 2013; Liu et al., 2014), while some authors associated the combination of this family and Prevotellaceae with a higher rate of reproductive inflammatory conditions (Mändar et al., 2015). In any case, the vast majority of human studies leave the presence of Porphyromonadaceae in the background, as its percentage is usually less than that of other families.

The other member from this same phylum is Prevotellaceae, traditionally defined as a natural component of vaginal, oral, cutaneous and digestive microflora. This family has been correlated with low semen quality by some authors (Weng et al., 2014), whereas others have found it to share a niche with healthy flora (Liu et al., 2014; Weng et al., 2014; Mändar et al., 2015). Our findings concur with the latter ones, as the animals in our study do not show clinical signs of disease and have a good semen quality. It is noteworthy to say that Bacteroidetes families scarcely appear in classical references. This is because this phylum is mostly composed of anaerobic gram-negative organisms. These bacteria are laborious to culture and, therefore, have been systematically omitted in culture-based microflora studies. Having pointed that, this family has neither been detected in the horse semen in other NGS studies (Al-Kass et al., 2020).

Regarding Corynebacteriaceae, it has been consistently defined as a natural component of seminal flora in humans (Liu et al., 2014; Weng et al., 2014; Mändar et al., 2015; Ivanov et al., 2009; Jarvi et al., 1996). This concurs with previous studies in the stallion (Al-Kass et al., 2020; Varela et al., 2018;, Althouse et al., 2010; Maasen and Christensen, 1995; Pickett et al., 1999; Varner et al., 1998). Other authors (Kiessling et al., 2008; Mändar, 2013) state that they are commensal bacteria that can become pathogenic when the flora unbalances or when there is a high activity of caspases (Ortega-Ferrusola et al., 2009).

The following four more common families belong to the Firmicutes phylum, Clostridia class: Peptoniphilaceae, Peptostreptococcaceae, Clostridiaceae and XI Family. Studies performed in humans show that these families are clearly rarer than in our case, excepting Peptoniphilaceae (Hou et al., 2013; Sanocka-Maciejewska et al., 2005). The same applies in the case of horses (Al-Kass et al., 2020). Interestingly, another class of this very same phylum, Bacilli, seems to be the most represented one in human semen, with families such as Lactobacillaceae, Staphylococcaceae and Streptococcaceae (Rando, 2012; Ng et al., 2010;, Hou et al., 2013; Weng et al., 2014; Varner et al., 1998; Fullston et al., 2015; Bromfield et al., 2014; Pasing et al., 2013; Rando and Simmons, 2015; Rodgers et al., 2013; Rota et al., 2011; Sharma et al., 2010). In our findings, these families were only represented at 0%, 0.17% and 0.22%, respectively. The only family related to these bacteria with a higher presence in our study was Aerococcaceae (1.23%), which has been associated with infertility cases in humans (Hou et al., 2013).

## Conclusion

In conclusion, the equine seminal microbiome is principally represented by Porphyromonadaceae, Peptoniphilaceae, Corynebacteriaceae and Prevotellaceae. A high inter-subject variability is also observed. Our results concur with the ones found in other studies (Al-Kass et al., 2020). However, they differ from studies made in other species (Hou et al., 2013; Weng et al., 2014; Rosenfeld et al., 2018; Javurek et al., 2016; Serrano et al., 2020). Further studies are needed to fully characterise the natural flora composition of stallion reproductive tract.
